# A New System of Skip-Lot Sampling Plans including Resampling

**DOI:** 10.1155/2014/192412

**Published:** 2014-01-21

**Authors:** Saminathan Balamurali, Muhammad Aslam, Chi-Hyuck Jun

**Affiliations:** ^1^Departments of Mathematics, Kalasalingam University, Krishnankoil 626190, India; ^2^Department of Statistics, Forman Christian College University, Lahore 54000, Pakistan; ^3^Department of Industrial and Management Engineering, POSTECH, Pohang 790-784, Republic of Korea

## Abstract

Skip-lot sampling plans have been widely used in industries to reduce the inspection efforts when products have good quality records. These schemes are known as economically advantageous and useful to minimize the cost of the inspection of the final lots. A new system of skip-lot sampling plan called SkSP-R is proposed in this paper. The performance measures for the proposed SkSP-R plan are derived using the Markov chain formulation. The proposed plan is found to be more efficient than the single sampling plan and the SkSP-2 plan.

## 1. Introduction

The acceptance sampling plans have been widely used in industries for maintaining the high quality level of the product at the minimum inspection cost [1, 2]. According to Taylor, [3] “one should follow this approach if you are uncertain of knowing how much sampling or inspection will be conducted on a day-by-day basis.” Deros et al. [4] discussed the application of these schemes in electrical and electronic products. An acceptance sampling plan helps the industrialists to yield high quality assurance. Various types of acceptance sampling plans like single sampling plan, double sampling plan, multiple sampling plan, sequential sampling plan, resampling schemes, repetitive group sampling plan, group acceptance sampling plan, and skip-lot sampling plans have been proposed for the attributes (just classifying the products as good or bad) and variables (measurement data) quality characteristics. Acceptance sampling plans provide the decision (accept or reject) about the submitted lot on the basis of sample information selected from the lot. Therefore, there is a chance of rejecting a good lot (producer's risk) and accepting a bad lot (consumer's risk). Therefore, new acceptance sampling plans are designed to minimize these two risks.

The skip-lot sampling plan (SkSP) provides a smaller sample size for the inspection purpose as compared to the single sampling plan. Skip-lot sampling plans have been widely used in industries to reduce the inspection cost. As pointed out by Hsu [5], the skip-lot sampling schemes are economically advantageous and useful to minimize the cost of the inspection of the final lots. The idea of SkSP-1 was originally introduced by Dodge [6–8]. The applications of SkSP-2 were discussed by Perry [9, 10]. Procedures and tables for the selection of the parameters of SkSP-2 plan indexed by various quality indices are available in the literature (see, e.g., [11]). Aslam et al. [12] studied the properties of SkSP-2 plan and developed tables for the selection of optimal parameters under binomial model. Balamurali and Subramani [13] investigated SkSP-2 plan with double sampling plan as the reference plan under the application of binomial model. The details and applications of the skip-lot sampling plans can also be seen in MIL-STD 105D [14], Okada [15], Stephens [16], Schilling [2], Parker and Kessler [17], Bennett and Callejas [18], Hsu [5], Carr [19], Cox [20], Liebesman and Sperstein [21], Reetz [22], ANSI/ASQC Standard A2-1987 [23], Liebesman [24], Duffuaa et al. [25], Cao and Subramaniam [26], and Aslam et al. [27, 28]. Balamurali and Jun [29] developed a new system of skip-lot sampling plan of type SkSP-V and investigated its properties.

The lot resubmission using resampling has been implemented in developing new sampling plans. Govindaraju and Ganesalingam [30] have proposed an attribute sampling plan which can be applied in situations where resampling is permitted on lots not accepted on the original inspection. They have derived the performance measures of the resampling scheme having single sampling attributes plan as the reference plan. Wu et al. [31] developed a variables sampling plan for resubmitted lots based on a process capability index. Aslam et al. [27, 28] also utilized the idea of resubmission in proposing a new sampling plan. So, it is expected that a skip-lot sampling plan can be more efficient if the idea of resampling concept is incorporated.

A new system of skip lot sampling plan utilizing resampling concept, denoted by SkSP-R, is given in Section 2. The performance measures for this SkSP-R plan are derived using the Markov chain approach and detailed in Section 3. The advantages and the properties of the proposed plan are given in Section 4.

## 2. SkSP-R Skip-Lot Sampling Plan

The SkSP-R plan is a new system of skip-lot sampling procedure, based on the principles of continuous sampling plans and resampling scheme for the quality inspection of continuous flow of bulk products. The SkSP-R plan uses the reference plan similar to the SkSP-2 plan of Perry [9]. The operating procedure of the SkSP-R plan is as follows, which is extension to the one in Balamurali and Jun [29].

1. Start with the normal inspection using the reference plan. During the normal inspection, lots are inspected one by one in the order of being submitted.
2. When *i* consecutive lots are accepted on the normal inspection, discontinue the normal inspection and switch to the skipping inspection.
3. During the skipping inspection, inspect only a fraction *f* of lots selected at random. The skipping inspection is continued until a sampled lot is rejected.
4. When a lot is rejected after *k* consecutively sampled lots have been accepted, then go for resampling procedure for the immediate next lot as in step 5 given below.
5. During resampling procedure, perform the inspection using the reference plan. If the lot is accepted, then continue the skipping inspection. On nonacceptance of the lot, resampling is done for *m* times and the lot is rejected if it has not been accepted on (*m* − 1)st resubmission.
6. If a lot is rejected on resampling scheme, then immediately revert to the normal inspection in step 1.
7. Replace or correct all the nonconforming units found with conforming units in the rejected lots.

**Figure 1:**
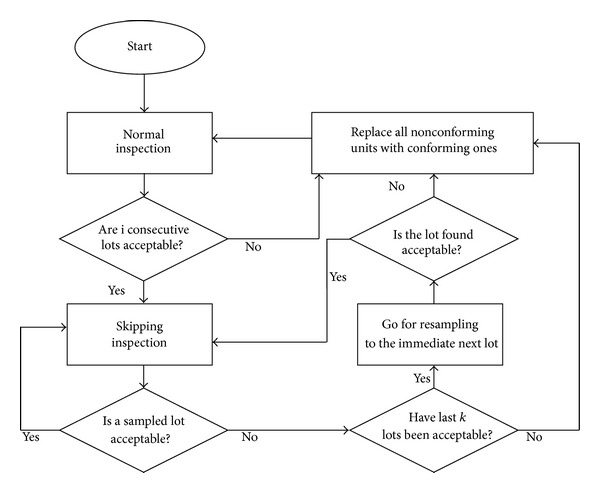
Operation of proposed skip-lot plan.

The operation of the proposed plan is depicted by a flow diagram in Figure 1.

The proposed plan involves the reference plan and four parameters, namely, *f*  (0 < *f* < 1), the fraction of lots inspected in skipping inspection mode, *i*, the clearance number of normal inspection, *k*, the clearance number of sampling inspection, and, *m*, the number of times the lots are submitted for resampling. In general, *i*, *k*, and *m* are positive integers. So, the plan is designated as SkSP-R (*f*, *i*, *k*, *m*). Govindaraju and Ganesalingam [30] recommended the use of *m* = 2 for their sampling plan. In this paper, we also consider *m* = 2 for numerical examples of the proposed plan.

## 3. Performance Measures of Proposed SkSP-R Plan

**Figure 2:**
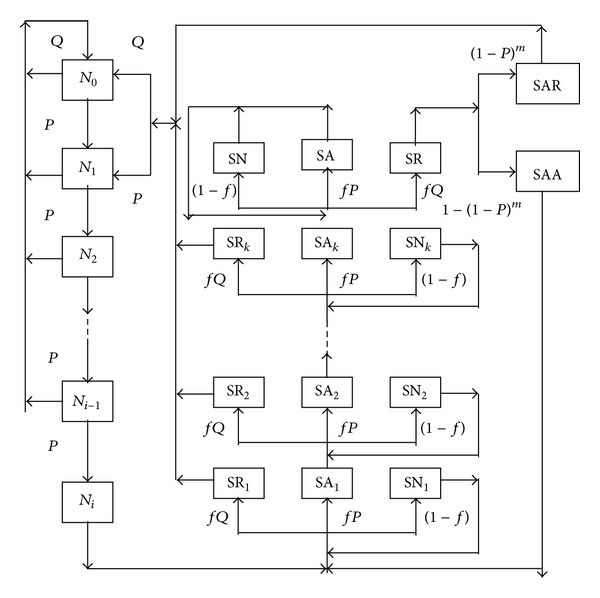
States and transitions of the proposed skip-lot procedure.

A finite state space can be defined and the transition among states can be modeled by a Markov chain. The state transition diagram having transition probabilities is shown in Figure 2, where the states are defined similarly as in Balamurali and Jun [29]. Here, *P* is the acceptance probability of a single lot under the reference plan and *Q* = 1 − *P*. The following are new states which did not appear in Balamurali and Jun [29]:

 SAR: state that a lot is rejected on the resampling procedure,
 SAA: state that a lot is accepted on the resampling procedure.

Various performance measures can be considered by deriving the limiting probabilities of the above Markov chain. The derivation method is very similar to the one in Balamurali and Jun [29], so it is omitted here. We consider the following measures of interest.

(1) The average number or the expected number of lots inspected under the normal inspection is
  (1) $$\begin{array}{c} U=\frac{\left(1-P^{i}\right)\left(1-P^{k}\left(1-Q^{m}\right)\right)+QP^{i+k}}{QP^{i}}, \end{array}$$
  where *Q* = 1 − *P*.
(2) The average fraction or the expected fraction of total submitted lots inspected in the long run is
  (2) $$\begin{array}{c} F=\frac{f+fQP^{i+k}-fP^{k}\left(1-P^{i}\right)\left(1-Q^{m}\right)}{f\left(1-P^{i}\right)\left[1-P^{k}\left(1-Q^{m}\right)\right]+P^{i}\left(1+fQP^{k}\right)}. \end{array}$$
(3) The probability of acceptance under the SkSP-R plan is
  (3) Pa(p) =fP+(1−f)Pi+fPk(Pi−P)(1−Qm)f(1−Pi)[1−Pk(1−Qm)]+Pi(1+fQPk).
(4) The average sample number (ASN) is
  (4) ASN(p) =nf+nfQPi+k−nfPk(1−Pi)(1−Qm)f(1−Pi)[1−Pk(1−Qm)]+Pi(1+fQPk).
(5) The average total inspection (ATI) is
  (5) ATI(p)=([n+(N−n)(1−P)]×[f+fQPi+k−fPk(1−Pi)(1−Qm)])×(f(1−Pi)[1−Pk(1−Qm)]+Pi(1+fQPk))−1.

## 4. Advantages of SkSP-R Plan

**Figure 3:**
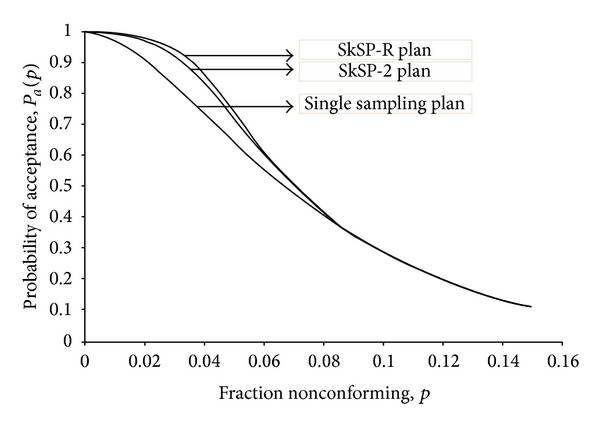
Operating characteristic (OC) curves.

In this section, we will compare the proposed plan with SkSP-2 and the single sampling plan which is the reference sampling plan in terms of probability of acceptance (*Pa*), average sample number (ASN), and average total inspection (ATI). We considered the SkSP-R plan with parameters *f* = 1/5, *i* = 6, *k* = 3, and *m* = 2, the SkSP-2 plan with *f* = 1/5 and *i* = 6, and the single sampling plan with parameters *N* = 1000, *n* = 25, and *c* = 1.  Figure 3 depicts the OC curves of three plans. From Figure 3, we can see that the proposed plan provides the higher probability of acceptance of the lots than the other two plans when the fraction nonconforming is smaller than 0.08. This indicates that the SkSP-R plan is useful for good quality level even though the OC curves of three plans coincide when the fraction nonconforming is >0.08.

**Figure 4:**
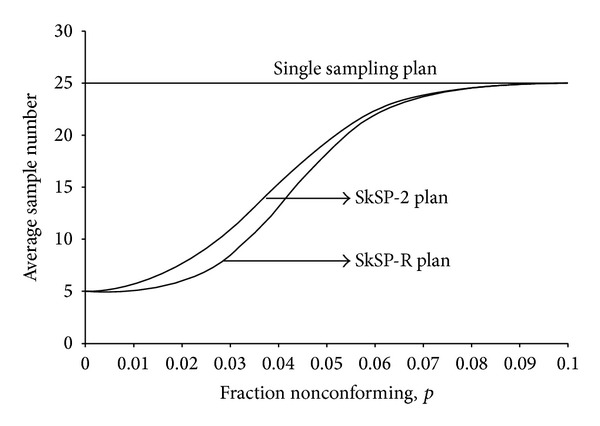
Average sample number (ASN) curves.

Figure 4 shows three curves of ASN for three sampling plans. From Figure 4, it can be noted that the proposed SkSP-R plan provides the smaller ASN as compared to SkSP-2 when *p* < 0.08, that is, for good quality level.

**Figure 5:**
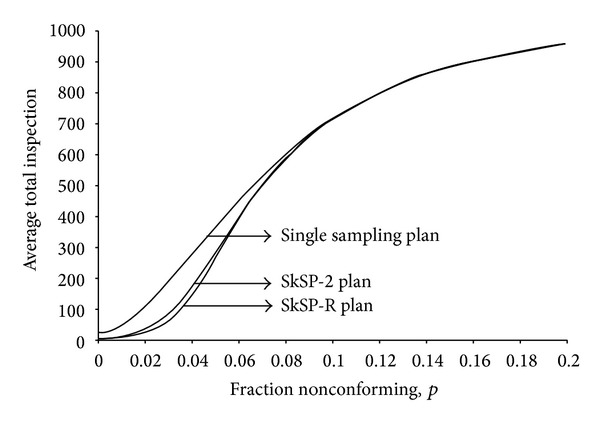
Average total inspection (ATI) curves.

In Figure 5, we see the three ATI curves for various values of *p*. Again, we can find that the proposed plan is better than the other two existing sampling plans in terms of ATI. The proposed plan provides smaller ATI than the other two sampling plans when the fraction nonconforming is <0.08.

**Example 1**

Suppose that an industrial engineer wants to apply the proposed plan for the inspection of the finished products. Suppose that the fraction nonconforming for this product (*p*) is 0.02. He is interested in looking for probability of acceptance, ASN, and ATI for the product under inspection. The probability of acceptance from the single sampling plan when *p* = 0.02 is almost 96%, from SkSP-2 is almost 98%, and from the proposed plan the lot acceptance probability is almost 99%. Therefore, the proposed plan is efficient in minimizing the chance of rejection of good lot. At *p* = 0.02, the values of ASN are 5 from the proposed plan; ASN is 7 from the SkSP-2 and 25 from the single sampling plan. Again, the proposed plan requires the less values of ASN as compared to the existing sampling plans which cause the reduction of the cost of the inspection. The value of ATI is almost 2 from the proposed plan, 3 from the SkSP-2 plan, and almost 9 from the single sampling plan. Also, the proposed plan is better than the other two sampling plans for any combinations of *f*, *i*, *k*, and *m*. This can be further proved with the help of Table 1. Table 1 gives the OC, ASN, and ATI values for SkSP-2 and SkSP-R plans for some combinations of *i*, *f*, *k*, and *m* with single sampling plan with parameters *N* = 1000, *n* = 50, and *c* = 1 as the reference plan. From this table, the efficiency of the proposed plan is clearly understood.

## 5. Conclusions

A new skip-lot sampling plan called SkSP-R has been proposed in this paper. Various performance measures of interest of the proposed plan have been derived using the Markov chain model. The advantages of the proposed plan over SkSP-2 and the reference plan are discussed in terms of ASN, ATI, and OC curves. The proposed plan is found to be more efficient than these two sampling plans in terms of ASN and ATI. The proposed plan is also shown to provide better protection to the producer and consumer than the other two existing sampling plans. This result concludes that the proposed plan performs better at smaller quality levels, which is the most useful situation encountered in practical applications. For higher values of quality levels, the proposed plan is not much efficient. The proposed plan requires more sample size or more inspection when the quality is poor. The strength of the proposed plan lies in achieving smaller ASN at low fraction nonconforming in which case the other two sampling plans require larger ASN and ATI. Hence the proposed plan is more economical than the traditional single sampling plan and the SkSP-2 plan. The SkSP-R plan using the cost model will be considered as a future research.

## Figures and Tables

**Table 1:** Comparison of the SkSP-R plan with SkSP-2 plan and single sampling plan at the fraction nonconforming, *p* = 0.01.

Parameters				OC			ASN			ATI		
*f *	*i *	*k *	*m *	SSP	SkSP-2	SkSP-R	SSP	SkSP-2	SkSP-R	SSP	SkSP-2	SkSP-R
0.1	10	5	2	0.91056	0.98024	0.98661	50	11.046	7.724	134.963	29.816	20.848
0.1	6	3	2	0.91056	0.98541	0.98958	50	8.157	6.127	134.963	22.017	16.539
0.1	6	6	2	0.91056	0.98541	0.98852	50	8.157	6.642	134.963	22.017	17.929
0.2	10	5	2	0.91056	0.96516	0.97475	50	19.475	14.566	134.963	52.568	39.317
0.2	6	3	2	0.91056	0.97273	0.97966	50	15.244	11.955	134.963	41.148	32.269
0.2	6	6	2	0.91056	0.97273	0.97785	50	15.244	12.817	134.963	41.148	34.597
